# Retraction: Simultaneous sorption and reduction of U(vi) on magnetite–reduced graphene oxide composites investigated by macroscopic, spectroscopic and modeling techniques

**DOI:** 10.1039/d0ra90041a

**Published:** 2020-04-23

**Authors:** Wencai Cheng, Zhongxiu Jin, Congcong Ding, Maolin Wang

**Affiliations:** Key Laboratory of Biological Resource and Ecological Environment of the Ministry of Education, College of Life Sciences, Sichuan University Chengdu 610064 P. R. China mlwang@scu.edu.cn +86 551 5591310 +86 551 5592758; Institute of Plasma Physics, Chinese Academy of Sciences P.O. Box 1126 Hefei 230031 Anhui P. R. China dingc_3650483@163.com

## Abstract

Retraction of ‘Simultaneous sorption and reduction of U(vi) on magnetite–reduced graphene oxide composites investigated by macroscopic, spectroscopic and modeling techniques’ by Wencai Cheng *et al.*, *RSC Adv.*, 2015, **5**, 59677–59685, DOI: 10.1039/C5RA10451C.

The Royal Society of Chemistry, with the agreement of the authors, hereby wholly retracts this *RSC Advances* article due to concerns with the reliability of the data in the published article.

The SEM image in Fig. 1C duplicates data published in another publication, but reported as different materials.^1^

There are unexpected similarities in the XPS spectra presented for M1–rGO and M5–rGO in Fig. 2A.

Repeating fragments can be observed in the baseline of the XRD spectrum for GO presented in Fig. 2B, which indicate that it has been manipulated. In addition, the XRD spectrum for M5–rGO in Fig. 2B duplicates data published in ref. 1 and 2, but they have been reported as different materials.

Given the number and significance of the concerns about the validity of the data, the findings presented in this paper are no longer reliable.

Signed: Wencai Cheng, Zhongxiu Jin, Congcong Ding and Maolin Wang

Date: 27^th^ March 2020

Retraction endorsed by Laura Fisher, Executive Editor, *RSC Advances*

## Supplementary Material
